# ECG Classification Using Orthogonal Matching Pursuit and Machine Learning

**DOI:** 10.3390/s22134960

**Published:** 2022-06-30

**Authors:** Sandra Śmigiel

**Affiliations:** Faculty of Mechanical Engineering, Bydgoszcz University of Science and Technology, 85-796 Bydgoszcz, Poland; sandra.smigiel@pbs.edu.pl; Tel.: +48-52-340-8346

**Keywords:** Machine Learning, orthogonal matching pursuit, Dictionary Learning, KSVD, Gabor dictionary, classification, ECG signal, PTB-XL database

## Abstract

Health monitoring and related technologies are a rapidly growing area of research. To date, the electrocardiogram (ECG) remains a popular measurement tool in the evaluation and diagnosis of heart disease. The number of solutions involving ECG signal monitoring systems is growing exponentially in the literature. In this article, underestimated Orthogonal Matching Pursuit (OMP) algorithms are used, demonstrating the significant effect of concise representation parameters on improving the performance of the classification process. Cardiovascular disease classification models based on classical Machine Learning classifiers were defined and investigated. The study was undertaken on the recently published PTB-XL database, whose ECG signals were previously subjected to detailed analysis. The classification was realized for class 2, class 5, and class 15 cardiac diseases. A new method of detecting R-waves and, based on them, determining the location of QRS complexes was presented. Novel aggregation methods of ECG signal fragments containing QRS segments, necessary for tests for classical classifiers, were developed. As a result, it was proved that ECG signal subjected to algorithms of R wave detection, QRS complexes extraction, and resampling performs very well in classification using Decision Trees. The reason can be found in structuring the signal due to the actions mentioned above. The implementation of classification issues achieved the highest Accuracy of 90.4% in recognition of 2 classes, as compared to less than 78% for 5 classes and 71% for 15 classes.

## 1. Introduction

Cardiovascular disease (CVD) is a term for disorders related to the heart and blood vessels. According to statistics released by the American Heart Association in 2019, CVDs have become the dominant global cause of death. In 2016, there were over 17.6 million deaths (31% of global deaths), estimated to reach 23.6 million in 2030.

In clinical diagnostics, the electrocardiogram (ECG) is the most commonly used tool to assess cardiovascular function. The choice of ECG is based on its widespread availability, as well as its non-invasive nature, repeatability, and low cost of the exam. The idea of ECG measurement is to analyze the electrocardiographic signal, which reflects the change in electrical potential generated by the heart during each work cycle. During the ECG test, the frequency of contractions is determined, thus showing abnormal heartbeat rhythm and activity. This test helps diagnose many heart diseases that damage the function of the heart muscle, including arrhythmia, myocardial infarction, and coronary artery disease. Early detection helps prevent complications, such as an increased risk of stroke or sudden death.

Over the past decade, numerous attempts have been made to identify the ECG signal. This has been possible mainly due to the availability of large, public open-source ECG datasets. The literature indicates the application of various approaches to ECG signal classification. Existing ECG signal classification models can be divided into two main categories: classical methods and deep learning methods. Many proposed approaches explore the Accuracy of classification algorithms, such as Support Vector Machines (SVM), Naive Bayes classifier, k-nearest neighbors algorithm (kNN), Decision Trees (DT), and group classifiers. A common aspect of these algorithms is the need to extract features from the input ECG signal. These features are multimodal, e.g., temporal, frequency, and statistical. The magnitude of these features is of variable importance in recognizing different classes of arrhythmias and is used to train Machine Learning (ML) algorithms.

The success of ECG classification using the classical ML method depends largely on selecting features, which must be carefully designed for different algorithms. In addition, the selected input dataset for which the classification process is also performed has a great influence. The second approach is based on deep learning techniques, which are increasingly used in computer-aided diagnosis of almost all diseases. Common deep learning networks used in ECG signal analysis are Convolutional Neural Networks (CNN) and Recurrent Neural Network (RNN), as well as Long Short Term Memory (LSTM) and their combinations.

Most classification studies are performed using the MIT-BIH Arrhythmia database and PTB Diagnostic ECG. Classification is usually performed using two or five classes of arrhythmias. For classical methods, SVM classifiers [1,2,3] combined with genetic algorithms [4], Wavelet Transform (WT) [5], or Discrete Wavelet Transform [6,7] have been used for most of them. The evaluation metric was most often Accuracy (ACC), for which the results took values of 91–93% up to five classes of arrhythmias and above five classes, with values between 95.92 and 99.66%. The authors also used models based on k-NN algorithms [7,8], taking into account prior extraction of morphological features of QRS complexes and Decision Tree (DT) algorithms [9]. In the area, five to seven different arrhythmias were subjected to these classifications, yielding an ACC of 99%. Convergent procedural scenarios are noticeable for the deep learning network. The authors undertook classifications of a similar number of arrhythmia classes. Mostly they used the CNN model [10,11]. The authors of the work [12] implemented a 1-D CNN to combine the feature extraction and classification process. A similar approach was used in the article [13], limited to classifying two arrhythmia classes, focusing on myocardial infarction detection. In [14], the authors used two different CNNs trained with 2-s and 5-s segments of ECG data to classify atrial fibrillation, atrial flutter, ventricular fibrillation, and normal rhythms. Improving the Accuracy in classification was undertaken by the authors of the article [15] using Short-Time Fourier Transform (STFT) and Stationary Wavelet Transform (SWT) to obtain 2D CNN. A combination of CNN with LSTM was presented by the authors of the article [16] for detecting five types of heartbeats, relying on variable-length ECG segments for feature generation. Continued work using the LSTM model was proposed by the authors of the article [17,18], undertaking the classification of two and eight classes of arrhythmias, respectively. An Accuracy of 99% was gained when they focused their research on aspects including atrial fibrillation. The new RNN architecture model has been successfully used to classify five types of ECG beats [19,20].

From the application perspective, ECG signal classification is important in remote patient monitoring devices. Their development and diffusion promote the prevention and treatment of cardiovascular diseases. Mobile solutions, i.e., small and discrete devices for long-term ECG monitoring, are associated with limitations. Especially when their purpose is to measure, analyze, archive, and transmit real-time data containing clinical information. This area was successfully recognized during the SARS-CoV-2 pandemic (COVID-19).

The correct interpretation of ECG signals is complex and clinically challenging, and misinterpretation can result in inappropriate treatment. Recommendations for standardization and interpretation of ECG are well known. However, the ubiquity of this test and the transition from analog to digital recordings have affected its detailed interpretation. Traditional approaches have increasingly focused on memorizing the morphological patterns of individual components of the ECG signal and associating them with a disease symptom. The idea seems to shift to automatic analysis of ECG signal fragments with the simultaneous classification of disease entities.

The process of diagnosing heart disease uses the information contained in electrocardiographic signals. The starting point in the evaluation of the ECG is the heart rate and the type of rhythm. The former is regulated by the heart rate and is related to the rate at which another follows one wave of the heart beat. The heart rhythm is the pattern in which the heart beats. It can be described as regular or irregular, fast or slow. A normal heart rhythm is called sinus rhythm. Its rate corresponds to the pulse, and accurate interpretation requires the evaluation of electrocardiographic signals. Various cardiovascular diseases can be detected with the help of the widely used electrocardiogram. Several parameters have clinical significance in the ECG, including PR interval, QRS complex, ST segment, and QT interval.

The authors often emphasized that data from single, small, or relatively homogeneous datasets, further limited by the small number of patients and rhythm, prevented the creation of reliable algorithms in Machine Learning models. To some extent, the PTB-XL database [21,22], for which multi-class classification work is already known [23,24,25], has become a solution to the problem of data inaccessibility.

This study aimed to find the best possible classifiers of classical Machine Learning methods for disease entities belonging to 2, 5, and 15 classes of heart disease. In addition, a new method for R-wave determination and QRS complex extraction was used in this study. This method uses a 12-lead signal for which an estimate of the R-peak position is generated using R-wave detection [23,24]. In this article, we used the Feature Selection Method approach [26] to perform the study in steps. The research was based on finding the optimal parameters from predefined parameters. Each stage was carried out for different classifier models, input data, dictionaries, and parameters, and four aggregation methods were developed. For this purpose, it was proposed to study nine classical Machine Learning classifiers using the Orthogonal Matching Pursuit algorithm.

This article is organized as follows. After the introduction, Section 2 presents the research methodology. The characteristics of the databases and the methods used in the article are discussed. Then, the feature detection from ECG signal and the application of classical Machine Learning models are specified. Implementation details and experimental results are described in Section 3. The conclusion and discussion are given after that.

## 2. Materials and Methods

Based on Feature Selection Methods [26], the different classification steps were planned. The study aimed to find the optimal classification model for 2, 5, and 15 classes related to heart disease. The number of classes should be interpreted as follows: 2 classes—NORM class and others from PTB-XL database, 5 classes—disease classes from PTB-XL database, and 15 classes—subclasses of diseases from PTB-XL database.

**Figure 1:**
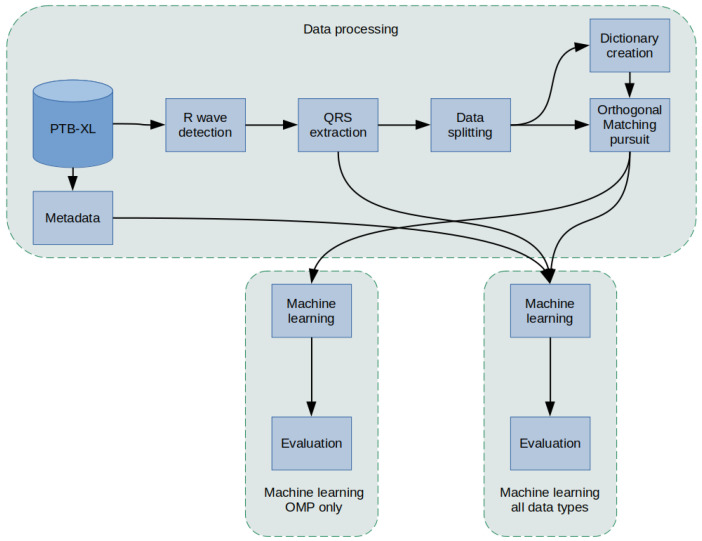
General overview diagram of the method.

The methodology used in this article was as follows (Figure 1): a PTB-XL dataset containing labeled 10-s ECG signal records was used for the study. First, the records in the database were filtered. Then, in the raw signal, R peaks were labeled and segmented so that there was precisely one QRS complex in each segment. In the next step, the data were divided into training data, validation, and test data (using cross-validation) and data for Dictionary Learning. In the next step, the dictionaries were created. Then, an Orthogonal Matching Pursuit operation was performed for the training data, resulting in the coefficients. Extracted QRS and coefficients were input for classifiers of 2, 5, and 15 heart disease classes. In the last step, an evaluation was conducted. The effectiveness of the proposed network methods was evaluated.

### 2.1. PTB-XL Dataset

In this study, all ECG data used are from the PTB-XL dataset [21,22]. The PTB-XL database is a clinical ECG dataset adapted for evaluating Machine Learning algorithms. Initially, PTB-XL consists of 21,837 records, corresponding to 12-lead ECG recordings. Each ECG signal is 10 s long and annotated by cardiologists. PTB-XL data are balanced by gender. The database contains 71 heart disease types with 5 relevant classes: normal ECG (NORM), myocardial infarction (CD), ST/T change (STTC), conduction abnormalities (MI), and hypertrophy (HYP).

**Figure 2:**
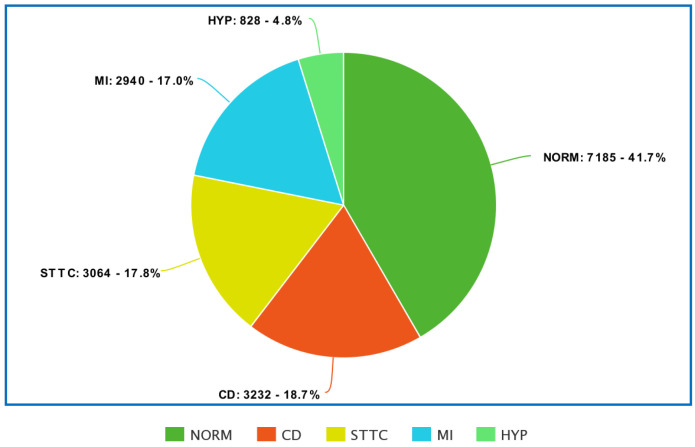
Distribution of PTB-XL database data by classes.

**Figure 3:**
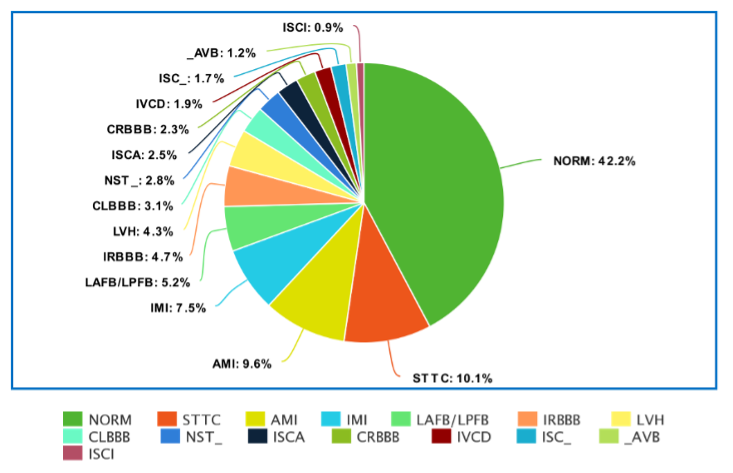
Distribution of PTB-XL database data by subclasses.

Figure 2 and Figure 3 show the detailed distribution of classes and subclasses used in the study. For Figure 2, the data include the number and the percentage of records. In contrast, for Figure 3, we are limited to the percentage of subclasses only.

### 2.2. Data Filtering

PTB-XL contained 21,837 ECG records. However, not all records have labels (assigned classes), and not all assigned classes have a specific 100% confidence (in terms of assigned medical diagnosis). For this reason, both cases were filtered from the original dataset. Each record has a specific class and subclass, defining cardiovascular disease. Records with less than 100 subclasses were also filtered out. This yielded 17,011 records, each belonging to one of 5 classes and one of 15 subclasses. For this study, it was decided to use ECG records with a sampling rate of 500 Hz.

### 2.3. R-Peak Detection

The classification studies were preceded by detecting features from the ECG signal. P wave, QRS complex, and T wave are its main components. The QRS complex was considered the leading one, for which the R-peak detection algorithm was developed. For R-peak detection, it was decided to investigate well-known detectors, such as: Hamilton [27], Two Average [28], Stationary Wavelet Transform [29], Christov [30], Pan-Tompkins [31], and Engzee [32] with modification [33]. For a better illustration of the developed algorithm, Listing A1 shows its implementation written in Python.

**Figure 4:**
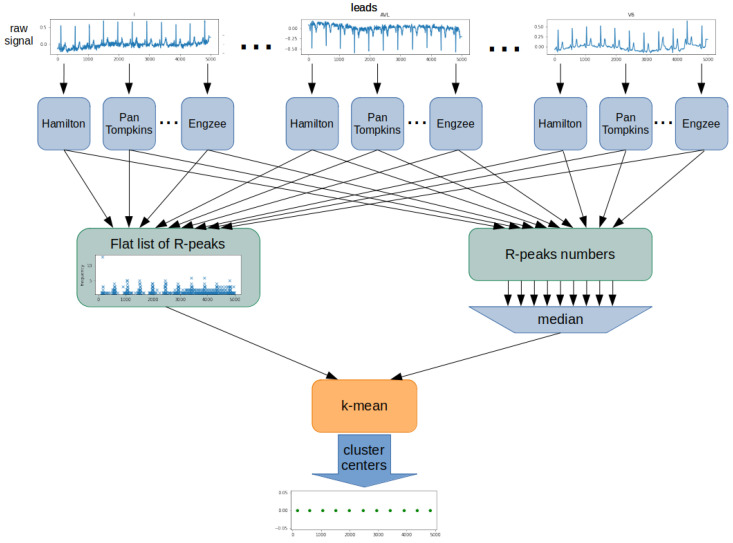
Block diagram of the proposed R-wave detection algorithm.

The proposed algorithm was based on determining the position and number of R-waves using all detectors for each ECG lead (Figure 4). The result was a list of detected R-peaks for each detector and lead. From this, a flat list of R-peak numbers was created, including all leads and detectors. Next, the algorithm determined the number of R-peaks in the examined ECG signal. For this purpose, the list’s median containing all counted R-peak was used. The last step was to determine the position of each of the R-peaks. The k-mean algorithm was used for this purpose. A flat list of R-peaks is used as training data for the k-mean algorithm. A determined R-peaks number is used as a k-value. Cluster centers of the k-mean algorithm are the determined R-peak positions.

Similar approaches applying different detectors for the Wavelet Transform examples and a variable number of leads have been proposed in [34]. The proposed approach combines three well-known algorithms operating on 1-lead ECG: Christov detectors, Pan-Tompkins, and Discrete Wavelet Transform. The aim of this procedure, as in the present work, was to obtain the best possible combination of different detectors for the highest Accuracy of R-wave detection.

This article’s test to evaluate the Accuracy of R-wave detection is presented in Appendix B. The tests were related to both 1-lead ECG signal and 12-lead ECG signal. The combination of the Two Average, Christov, and Engzee detector for the research methodology was further used due to the Original Algorithm.

### 2.4. QRS Extraction

The determined positions of the R-peaks were used to extract the segments containing the QRS complexes. For a 10-s signal fragment, this operation consisted in determining the midpoint of the segments between consecutive R-peaks. The first and last segments obtained in this way were discarded. With this procedure, the R-peaks and thus the QRS complex would always be in the middle of the segment.

### 2.5. Description of the Implemented Method

**Figure 5:**
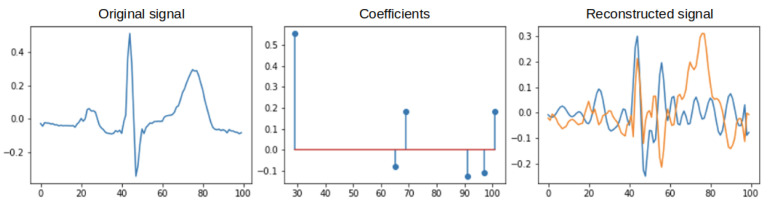
Example input ECG signal (original), non–zero coefficients, the signal after reconstruction.

**Figure 6:**
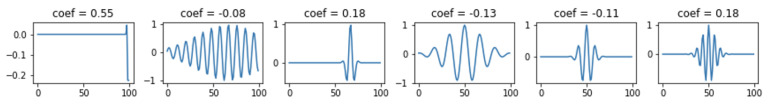
Atoms with non–zero coefficients, for example, signal decomposed using 6 non-zero coefficients.

**Figure 7:**
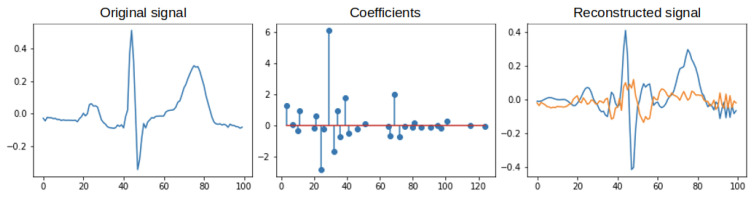
Operation of the OMP algorithm −30 non–zero coefficients.

**Figure 8:**
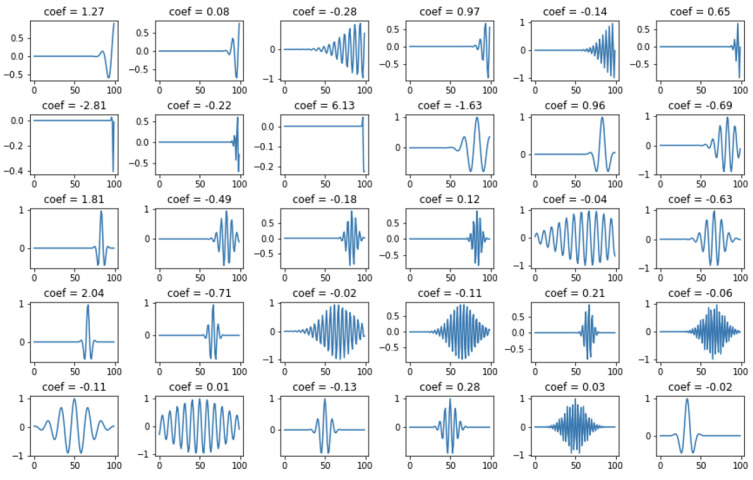
Atoms with non–zero coefficients, for example, signal decomposed using 30 non–zero coefficients.

Orthogonal Matching Pursuit was assumed to be the primary technique [35,36]. The Orthogonal Matching Pursuit (OMP) algorithm is an extension of the Matching Pursuit (MP) algorithm. The OMP algorithm, like MP, is based on a continuous search and matching of appropriate elements (atoms) of the dictionary that best reflect the desired features of the studied (original) signal. This process should maximize the correlation between an element from the dictionary and the rest of its part (residual) of the processed signal. The result of the OMP is a vector of coefficients. To give an idea of the process discussed above, Figure 5 shows an example of the original (input) signal, the coefficients obtained from its decomposition, and the signal after reconstruction and the residual (the difference between the original signal). The blue color indicates the signal after reconstruction, and the orange color indicates the residual. The decomposition was performed with 6 non-zero coefficients. Figure 6 shows the selected atoms with their coefficients. The expansion for this case is shown in Figure 7 and Figure 8, where 30 non-zero coefficients were used respectively.

### 2.6. Dictionary Created Using Dictionary Learning Technique

**Figure 9:**
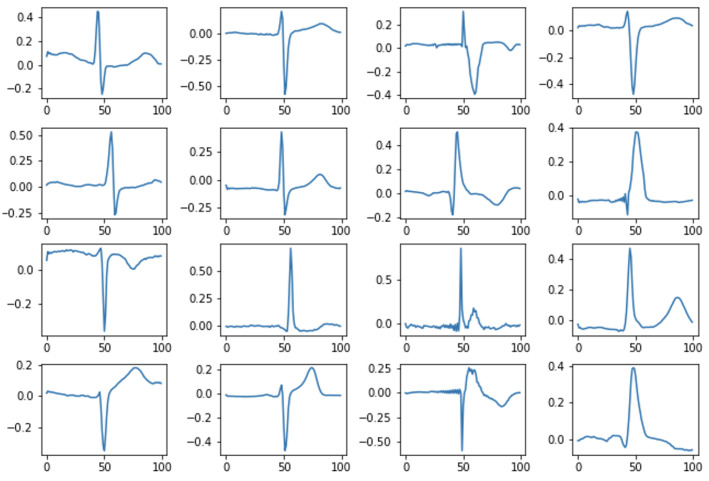
Example atoms of the DL dictionary.

Dictionary Learning dictionary creation is based on data [37]. The task of the algorithms is to find a dictionary of atoms that best represents a given signal type. Figure 9 shows an example of Dictionary Learning atoms.

### 2.7. Dictionary Created Using KSVD Technique

**Figure 10:**
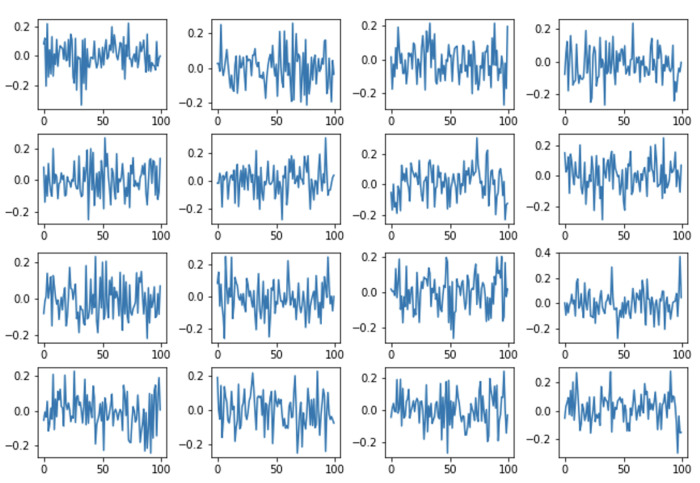
Examples atoms of the KSVD dictionary.

KSVD [38] is an algorithm from the Dictionary Learning group that performs Singular Value Decomposition (SVD) to update the dictionary atoms, one by one, and is a kind of generalization of the k-means algorithm. An example of KSVD dictionary atoms is shown in Figure 10.

### 2.8. Designed Machine Learning Algorithms

The following classifiers were examined: KNeighbors—k-Nearest Neighbors [39], DecisionTree—Decision Tree [40], RandomForest—Random Forest [41], SVC—Support Vector Machine [42], XGBoost, LGBM—LightGBM, MLP—Multi-Layer Perceptron [43], AdaBoost [44], and GaussianNB—Naive Bayesian Classifier.

**Figure 11:**
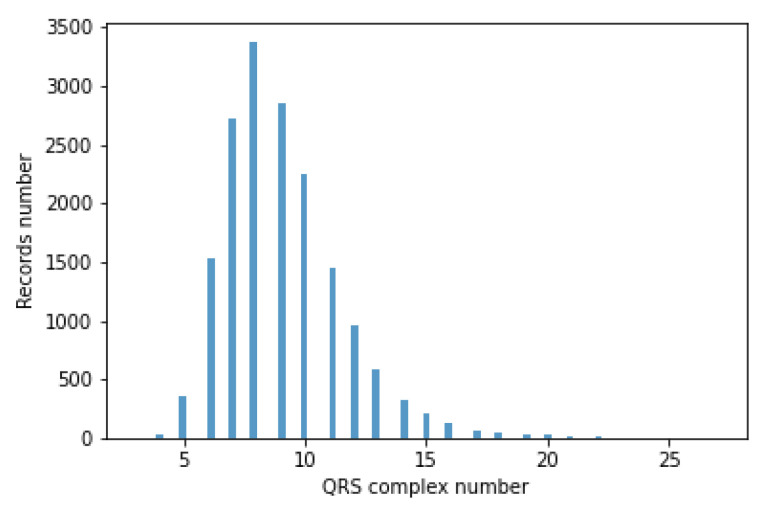
Histogram of the number of segments comprising the QRS complex.

The classifiers take a fixed-size data vector as input. Unfortunately, for different records, the number of QRS episodes varies (BPM varies), ranging from 3 to 27. This causes the flat data vector with a different number of QRS to have a variable size. Accordingly, QRS episode aggregation methods have been proposed. A histogram of the number of segments containing the QRS complex is shown in Figure 11.

The author’s 4 methods of aggregation, i.e., a grouping of episodes containing QRS complexes, were developed. In the rest of the article, the proposed aggregation methods are called Single, Mean, Max, and Voting.

The inputs of aggregation methods were ECG signal records, episodes containing QRS complexes extracted on their basis, and the result of the OMP algorithm, i.e., coefficients obtained from them. In each aggregation method, the model’s output is a prediction corresponding to the disease entities.

**Figure 12:**
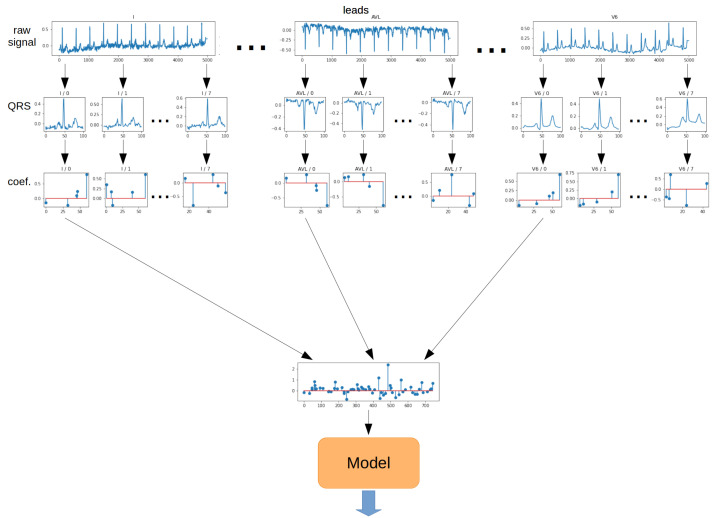
Aggregation method—Single.

The **Single method** is the most straightforward approach to QRS segment aggregation. The principle is to take a vector of coefficients obtained from the OMP algorithm for the first QRS segment from each lead. As a result, a 2-dimensional matrix was obtained. Then, such a matrix of coefficients was transformed into a 1-dimensional vector. The resulting vector was fed to the model input. The schematic for the Single method is shown in Figure 12.

**Figure 13:**
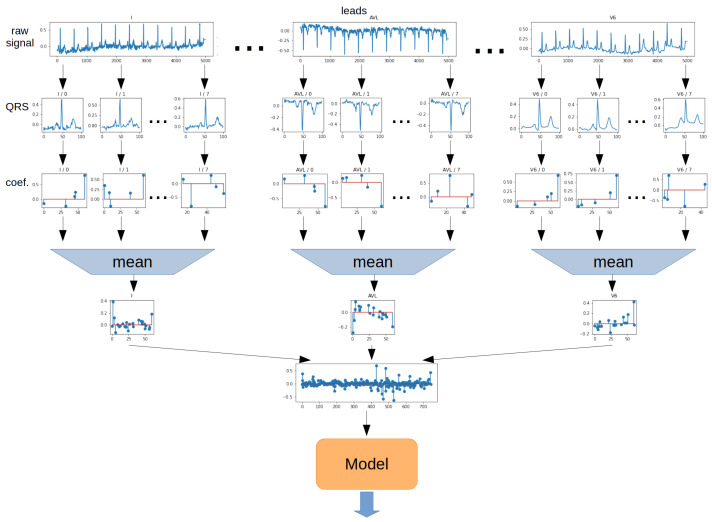
Aggregation method—Mean.

The **Mean method** involves determining the arithmetic mean of the values of each ratio for all QRS segments. The operation was performed separately for each lead. The 2-dimensional matrix was then transformed into a 1-dimensional vector. In the next step, the vector was the model input. The schematic for the Mean method is shown in Figure 13.

**Figure 14:**
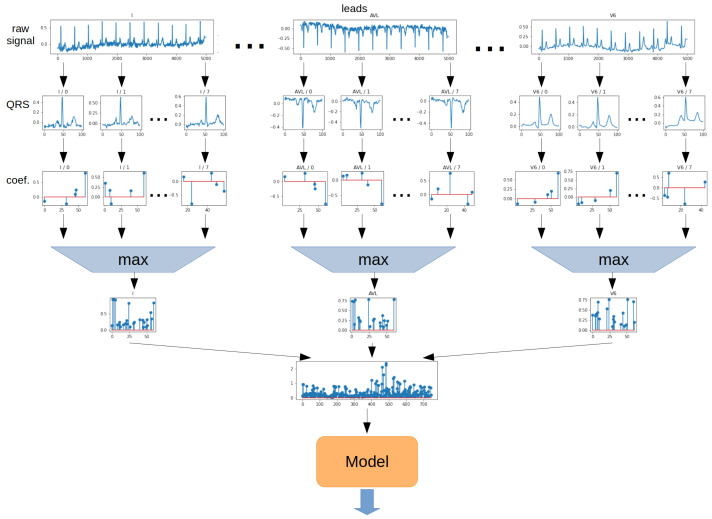
Aggregation method—Max.

The **Max method** determines the maximum from the absolute value of each ratio from all QRS episodes. The operation was performed separately for each lead. The 2-dimensional matrix was then transformed into a 1-dimensional vector. In the next step, the vector was the model input. The schematic for the Max method is shown in Figure 14.

**Figure 15:**
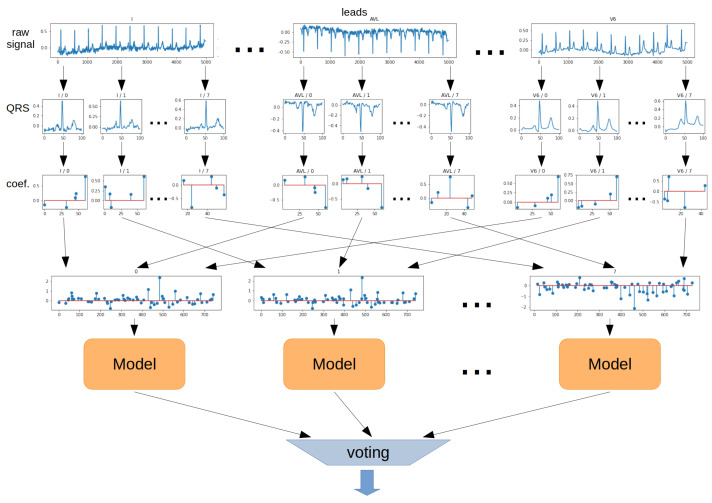
Aggregation method—Voting.

The **Voting method** involves training model consisting of all QRS episodes. For this purpose, for each QRS segment, a 2-dimensional coefficient matrix is transformed into a 1-dimensional vector and given to the model input. In contrast, prediction is performed for each QRS segment separately. In the next step, the arithmetic mean of the prediction probabilities derived from each QRS segment is determined, and the prediction is made on this basis. The schematic for the Voting method is shown in Figure 15.

### 2.9. Data Splitting

The following data were used for each record:

- Metadata (sex, age, BPM, resampling ratio);
- Segments containing QRS complexes;
- Coefficients from the OMP algorithm;
- Dictionary and its parameters, i.e., dictionary type (Dictionary Learning (DL), KSVD, Gabor), dictionary size (62, 125, 250, 500, 1000 elements), number of non-zero coefficients (5, 10, 20, 40);
- Aggregation methods: Single, Mean, Max, Voting.

Records were divided into training, validation, and test data in proportions of 70%, 15%, and 15%. To improve the quality of testing, non-exhaustive cross-validation was used. For this purpose, the split function was called with 3 or 5 different seeds. This means that all tests were repeated five times for different data splits.

### 2.10. Metrics

Models were evaluated using the metrics described below [45]. For simplicity of equations, specific acronyms have been created: TP—True Positive, TN—True Negative, FP—False Positive, and FN—False Negative.

The metrics used for network evaluation are:

- Accuracy: Acc=(TP+TN)/(TP+FP+TN+FN);
- Precision=TP/(TP+FP);
- Recall=TP/(TP+FN);
- F1=2·Precision·Recall/(Precision+Recall);
- Balanced Accuracy: BAcc=1/2·(TP/(TP+FN)+TN/(TN+FP)).

### 2.11. Used Tools

The computations were performed on a server equipped with 2 Intel Xeon Silver 4210R processors (192 GB of RAM), Nvidia Tesla A100 (40 GB RAM), and Nvidia Tesla A40 (48 GB RAM) GPUs. They were also performed on 5 servers, each of which was equipped with 2 Intel Xeon Gold 6132 processors (512 GB of RAM). In this research, Sklearn, Numpy, Pandas, and Jupyter Lab programming solutions were used.

## 3. Results

**Figure 16:**
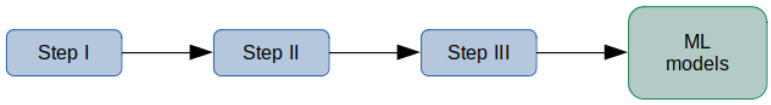
Steps of implementation of research related to classification.

The classifiers were evaluated by dividing them into three steps, as shown in Figure 16.

### 3.1. Step 1

Step 1—started with the initial selection of dictionaries and models. The study was conducted for classification based on five classes. The model input vector included the coefficients obtained from the OMP algorithm. Results were obtained for parameter combinations:

- Dictionary: Dictionary Learning (DL), Gabor, KSVD;
- Dictionary size: 62, 125, 250, 500 i 1000 elements;
- Number of non-zero coefficients: 5, 10, 20, 40;
- Aggregation methods: Single, Mean, Max;
- Classifier model: KNeighbors, DecisionTree, RandomForest, SVC, XGBoost, LGBM, MLP, AdaBoost, GaussianNB.

The created combinations were tested for three different seeds. The 1620 parameter combinations were analyzed. Table 1 summarizes the results of the classification calculations for all dictionary types and sizes, arranged according to decreasing values of the Accuracy metric (ACC). Correspondingly, Table 2 summarizes the results for each aggregation method and model. The tables show the averaged Accuracy (ACC), Precision, Recall, and F1 values.

### 3.2. Step 2

Step 2—tests were conducted for classifications of 2, 5, and 15 classes. The input vector included the coefficients obtained from the OMP algorithm. Results were obtained for combinations of parameters:

- Dictionary: Gabor, KSVD;
- Dictionary size: 125, 250, 500 elements;
- Number of non-zero coefficients: 5, 10, 20, 40;
- Aggregation methods: Mean, Voting;
- Classifier model: XGBoost, LGBM.

The created combinations were tested for five different seeds. The 96 parameter combinations were analyzed. Table 3, Table 4 and Table 5 summarize the results for each aggregation method and model, arranged according to decreasing values of the Accuracy metric (ACC). The tables show the averaged Accuracy (ACC), Precision, Recall, and F1 values.

### 3.3. Step 3

Step 3—testing was performed for classifications 2, 5, and 15. The input vector included coefficients obtained from the OMP algorithm, sections containing QRS complexes in the form of raw signal and metadata. Results were obtained for combinations of parameters:

- Input data type: signal, coef, meta, signal + coef, signal + meta, coef + meta, signal + coef + meta;
- Dictionary: Gabor, KSVD;
- Dictionary size: 125, 250 elements;
- Number of non-zero coefficients: 20, 40;
- Aggregation methods: Voting;
- Classifier model: XGBoost, LGBM.

The created combinations were tested for five different seeds. The 70 parameter combinations were analyzed. Table 6, Table 7 and Table 8 summarize the results for each aggregation method and model, arranged according to decreasing values of the Accuracy metric (ACC). The tables show the averaged Accuracy (ACC), Precision, Recall, and F1 values. The N/A designation means not applicable.

**Figure 17:**
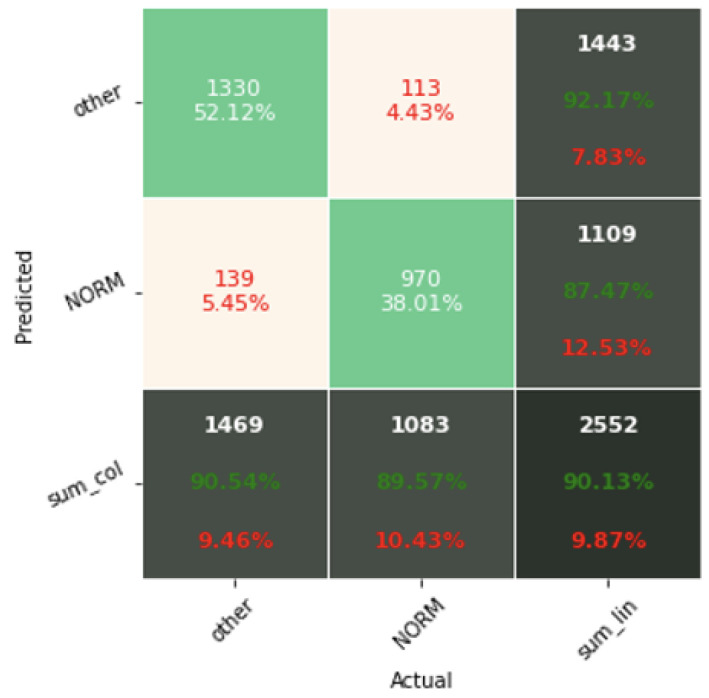
Confusion matrix of the best model in classification for 2 classes.

**Figure 18:**
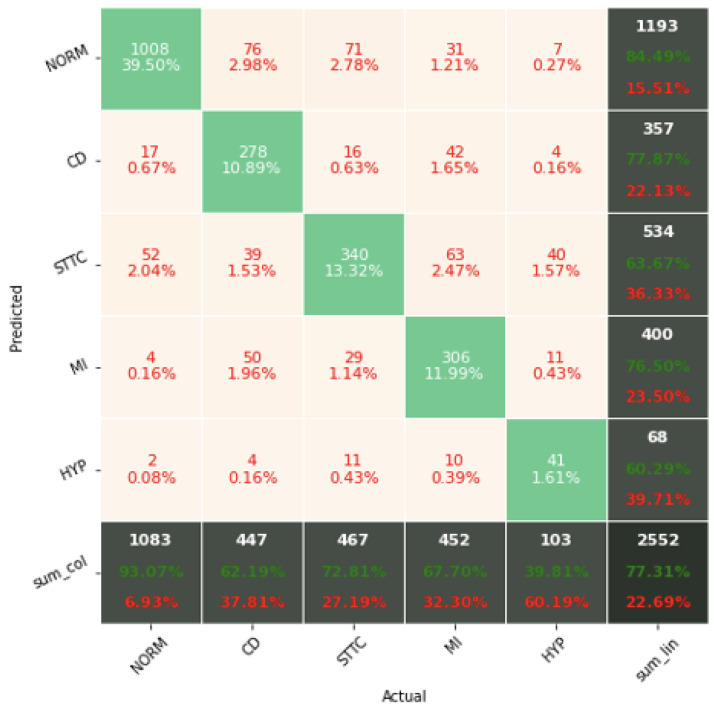
Confusion matrix of the best model in classification for 5 classes.

**Figure 19:**
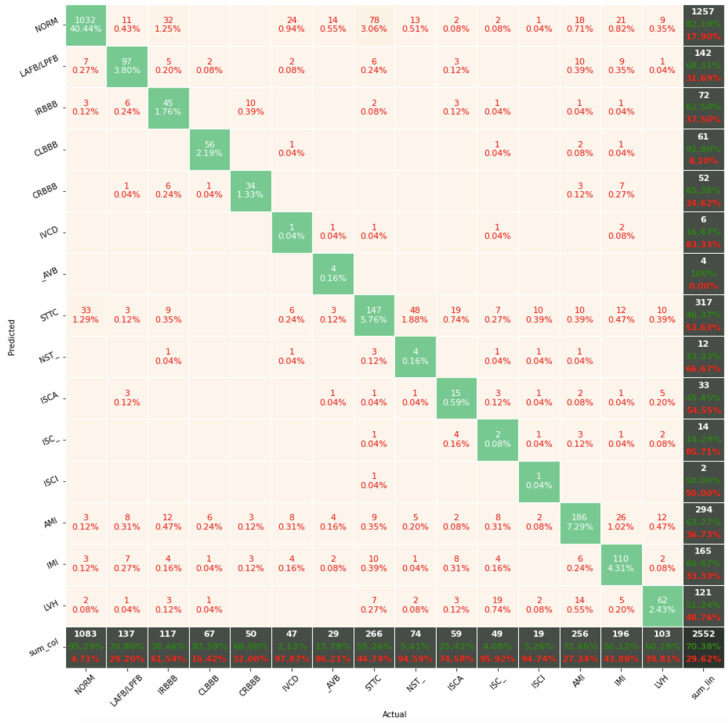
Confusion matrix of the best model in classification for 15 classes.

Figure 17, Figure 18 and Figure 19 present the confusion matrices from the evaluation on the test dataset. The confusion matrices generated in this article are for the first seed only, in order to limit their volume. For the other seeds they look similar. The confusion matrices include classification results for 2, 5, and 15 classes.

## 4. Discussion

The classification of an ECG signal is a complex issue, for which many obstacles limit the high Accuracy of the conducted studies. The studies and analyses need to integrate available methods with feature extraction techniques from electrocardiographic signals. Only in this approach is it possible to achieve the objectives at the highest possible level from the clinical perspective and not only because of non-medical diagnostics.

Although different results are available for ECG classification experiments, it is difficult to compare directly due to different classification schemes and evaluation metrics. Furthermore, one can see differences in the adopted classification objectives, which are not always aimed at obtaining the highest possible scores and often show differences when using different classification models. Nevertheless, the methodology proposed for this work achieved relatively good results for the PTB-XL database compared to other works. However, to give an idea of the current state of the art, it was decided to present related studies using different classifiers within other databases.

ECG signal classification is known primarily from articles involving the diagnosis of myocardial infarction, atrial fibrillation, or ventricular fibrillation. An equally wide range of articles relates to the general definition of arrhythmias. For example, articles in myocardial infarction classification [46,47] based on classical SVM-type classifiers are known from research for the PTB Diagnostic ECG Database or MIT-BIH Arrhythmia [48]. The test set results were ACC = 0.9958, ACC = 0.9874, and ACC = 0.976, respectively. Although they obtained high scores, the dataset dependencies remain uncertain for which, due to the small number of waveforms, it is possible that they used ECG signal segments from the same patient during model validation and testing. In addition, these results should not be interpreted as a classification for the two classes used in this study. The authors also chose to use models based on kNN algorithms [6,8], considering the previous extraction of QRS complexes and Decision Tree algorithms [9]. The evaluation metric most commonly used was Accuracy, for which results took values of 0.910–0.930 for five classes of arrhythmias and above five classes, values ranging from 0.9592 to 0.9966.

This work implemented classification for 2, 5, and 15 classes. Each step was carried out for different classifier models, input data, and aggregation methods. The input data were enriched with features derived from the Orthogonal Matching Pursuit algorithm, including different dictionaries and their parameters. The author’s algorithm for R-wave determination and QRS complex extraction was also evaluated.

For the purpose of this article, tests were performed to assess the computational complexity of the study. The results are summarized in Table A3, located in Appendix C. The measured times correspond to the model training and prediction steps on the validation and test sets. The experiment was performed for the Single aggregation method and using only OMP coefficient vectors’ given inputs. For most of the classifiers considered, the length of computation was more dependent on the size of the dictionary than the number of classes. One can see a differential increase in these times depending on the model. For example, for the Decision Tree model, as the size of the dictionary and the number of classes increased, the computation increased slightly. The situation looks different for the XBoost model, where the calculation times increase significantly. The longest calculation times were observed for the SVC classifier.

In evaluating cardiac classification algorithms, it is important to evaluate the R-peak detection algorithm. Based on the results obtained, the Two Average detector showed the highest Accuracy in R-peak detection in the 1-lead approach, whereas the Engzee detector showed the lowest. Different combinations for the 12-lead signal significantly influenced the results obtained. An improvement in the Accuracy of R-peak detection could be observed with the 12-lead approach to the signal under study. For example, Two Average detector in the classical approach achieved MAE = 0.389, and in that proposed for this study MAE = 0.270. This was due to the simultaneous consideration of all leads from the examined 10-segment ECG signal. The results were completely different when all detectors were selected simultaneously, for which the MAE evaluation metric was 0.367. Although, concerning the classical best approach, i.e., MAE = 0.389, it still obtained a higher score. The solution proposed for this article indicates that there is undoubtedly some optimal combination of detectors that provides the best results. In the case of analyzed ECG signals, the highest Accuracy of R-peak detection was obtained by combining the Two Average detector, the Christov detector, and the Engzee detector.

The experiments conducted showed that the results on the test data differ little from the results on the validation data. The values of the ACC classification metric for grades 2 and 5 remain higher than the tests in the work [23,24], regardless of the approach used. In the case of the work [25], they remain lower. The obtained Accuracy results for the classification of two and five classes achieved an Accuracy of 0.9023 and 0.7766, respectively. It is impossible to compare the classification for 15 classes, which has not been attempted so far by the authors of other works. The classification results for 15 classes achieved an Accuracy of 0.7079.

The problem of heart disease classification using classical models of Machine Learning methods was supported by underestimated Orthogonal Matching Pursuit (OMP) algorithms, showing the significant effect of concise representation parameters on improving the Accuracy of the classification process. Different combinations of dictionaries created for the operation of the OMP algorithm were investigated. Their optimal parameters were determined. The study shows that not only the type of dictionary is important. Its size and the number of non-zero coefficients are also important. The realized studies indicate that the hybrid system provides the highest ACC metric scores. For this system, the input data vector includes coefficients obtained from the OMP algorithm, segments containing QRS complexes in raw signal form, and metadata.

The RandomForest, XGBoost, or LightGBM classifiers proposed in this article are Decision Tree-based models designed to work with structured data. Thus, models cannot cope with unstructured data. This is the inability to recognize and detect shapes and displacements. It can be speculated that the approach proposed in this article, related to extraction of QRS complexes and resampling of the raw signal, contributes to locating the R-peak always in the same place and structures the data well enough for tree-based models to cope.

The realized experiments highlight the Accuracy of the proposed Voting aggregation method, for which the highest Accuracy results were obtained, regardless of the model or class size. A similar observation was noted in the comparison of dictionaries, where dictionaries created using Gabor functions were found to be the best. The performed experiments also emphasized the importance of using coefficients obtained from the OMP algorithm, for which the tested models obtained the highest Accuracy.

The confusion matrix analysis gives more possibilities to evaluate the obtained results. Classification Accuracy for two classes regardless of classifier type seems to be true. Although also, in this case, for subclasses with a small number of records, skipping occurs, which affects to some extent the skewness of the model. However, this explains the worse classification performance as the class size increases, i.e., 5 and 15 classes. What is particularly clear in Figure 19 is that a large part of the misclassification is caused by an imbalanced dataset. Classes with a low record number (such as IVCD or ISCI) are less frequently selected by the model. The situation is different for classes with many records (such as NORM or STTC). The model more often selects them. The NORM class is the most numerous, which makes it the best internalized by the model. It has the highest Precision and Recall values (green percentages on the bottom and right matrix bars). This is confirmed by the balanced Accuracy presented in Table 1, Table 2, Table 3, Table 4, Table 5, Table 6, Table 7 and Table 8.

If the aim of the work was to evaluate the results obtained in terms of statistical significance, it would be necessary to carry out Levenes test, Annova test, and Tukey-Hsd test. In the analyzed article, such an approach was carried out for step 3, considering this step as final. In the case of classification for 2, 5, and 15 classes, the results of Levenes test reached *p* > 0.05 and the results of Annova test reached *p* < 0.05. Realizing the comparison of different combinations of models with the use of Tukey-Hsd test, it can be noticed that statistically significant differences of the ACC metric for two classes are obtained above 1%, and respectively for 5 classes and 15 classes, −2.5% and 3.8%.

## 5. Conclusions

The issues corresponding to ECG signal classification were realized in the increasingly dynamic Machine Learning methods. The implementation of the classification issues in work achieved the highest Accuracy of 90% in recognizing 2 classes, whereas less than 78% for 5 classes and 71% for 15 classes. The research was undertaken on the recently published PTB-XL database, whose ECG signals were previously subjected to detailed analysis. Orthogonal Matching Pursuit algorithms were used, demonstrating the effect of concise representation parameters on improving the Accuracy of the classification process. Heart disease classification models based on classical classifiers were defined and investigated. Authors’ methods of aggregation of ECG segments containing QRS complexes were proposed. As a result, it was proved that the ECG signal subjected to algorithms of R-peak detection, QRS complexes extraction, and resampling performs very well in classification using Decision Trees. This is due to the structuring of the signal due to the actions mentioned above.

## Figures and Tables

**Table 1:** Summary of average metrics scores for each dictionary, class size = 5.

Position	Dictionary	Model-Method	ACC	Precision	Recall	F1	BACC
1	Gabor-1000-20	LGBM-Mean	0.736	0.692	0.613	0.634	0.613
3	KSVD-250-5	LGBM-Mean	0.734	0.688	0.621	0.642	0.621
4	KSVD-500-10	LGBM-Mean	0.733	0.691	0.613	0.635	0.613
5	Gabor -125-20	LGBM-Mean	0.733	0.685	0.619	0.639	0.619
7	KSVD-125-5	LGBM-Mean	0.731	0.691	0.614	0.636	0.613
9	Gabor -250-20	LGBM-Mean	0.73	0.688	0.607	0.629	0.607
25	DL-62-5	LGBM-Mean	0.726	0.677	0.601	0.623	0.601
31	Gabor -500-10	LGBM-Mean	0.725	0.685	0.604	0.625	0.603
32	KSVD-1000-10	LGBM-Mean	0.724	0.671	0.604	0.624	0.604
37	KSVD-62-10	LGBM-Mean	0.724	0.681	0.603	0.625	0.602
54	DL-125-5	LGBM-Mean	0.721	0.685	0.593	0.617	0.593
55	Gabor -62-40	LGBM-Mean	0.721	0.68	0.617	0.638	0.616
64	DL-250-5	LGBM-Mean	0.719	0.673	0.587	0.608	0.586
99	DL-500-5	LGBM-Mean	0.714	0.673	0.573	0.593	0.572
127	DL-1000-5	LGBM-Mean	0.708	0.653	0.568	0.587	0.568

**Table 2:** Summary of average metrics scores for each aggregation method and classifier model, class size = 5.

Position	Dictionary	Model-Method	ACC	Precision	Recall	F1	BACC
1	Gabor-1000-20	LGBM-Mean	0.736	0.692	0.613	0.634	0.613
12	Gabor-125-20	XGBoost-Mean	0.729	0.676	0.613	0.632	0.613
39	KSVD-125-10	SVC-Mean	0.724	0.673	0.611	0.632	0.611
60	KSVD-250-5	LGBM-Max	0.72	0.668	0.624	0.641	0.624
93	KSVD-500-5	XGBoost-Max	0.715	0.664	0.614	0.633	0.614
115	KSVD-500-10	SVC-Max	0.709	0.667	0.573	0.593	0.573
150	Gabor-125-20	Random Forest-Mean	0.703	0.658	0.555	0.574	0.554
159	KSVD-250-20	MLP-Mean	0.703	0.635	0.613	0.622	0.613
187	Gabor-125-20	LGBM-Single	0.699	0.645	0.588	0.607	0.588
222	Gabor-125-20	XGBoost-Single	0.696	0.638	0.578	0.597	0.578
265	DL-1000-10	MLP-Max	0.691	0.616	0.563	0.578	0.563
373	KSVD-125-5	SVC-Single	0.681	0.631	0.551	0.573	0.551
393	Gabor-125-10	Random Forest-Single	0.678	0.636	0.539	0.562	0.539
459	KSVD-250-5	Random Forest-Max	0.671	0.651	0.522	0.549	0.522
564	KSVD-62-5	MLP-Single	0.656	0.566	0.556	0.560	0.556
696	Gabor-500-40	KNeighbors-Mean	0.632	0.597	0.468	0.482	0.468
698	DL-1000-20	GaussianNB-Mean	0.631	0.519	0.495	0.502	0.494
718	Gabor-125-5	AdaBoost-Mean	0.628	0.534	0.527	0.521	0.526
825	KSVD-250-5	Kneighbors-Max	0.61	0.629	0.43	0.440	0.430
865	DL-1000-10	GaussianNB-Max	0.603	0.498	0.465	0.473	0.465
943	Gabor-125-10	AdaBoost-Max	0.586	0.489	0.499	0.478	0.499
950	Gabor-125-10	AdaBoost-Single	0.585	0.485	0.486	0.472	0.486
971	Gabor-125-5	Decision Tree-Mean	0.582	0.474	0.476	0.474	0.476
977	Gabor-125-10	Kneighbors-Single	0.581	0.551	0.414	0.428	0.414
1086	Gabor-125-20	Decision Tree-Single	0.56	0.458	0.458	0.457	0.458
1150	DL-250-20	GaussianNB-Single	0.548	0.449	0.417	0.411	0.416
1176	KSVD-1000-5	Decision Tree-Max	0.543	0.447	0.44	0.443	0.439

**Table 3:** Summary of average metrics scores for each data model, class size = 2.

Position	Dictionary	Model-Method	ACC	Precision	Recall	F1	BACC
1	Gabor-125-20	XGBoost-Voting	0.897	0.893	0.897	0.895	0.897
3	Gabor-125-20	LGBM-Voting	0.896	0.892	0.895	0.893	0.895
4	Gabor-125-20	LGBM-Mean	0.895	0.891	0.896	0.893	0.895
12	Gabor-125-20	XGBoost-Mean	0.892	0.888	0.892	0.889	0.892

**Table 4:** Summary of average metrics scores for each data model, class size = 5.

Position	Dictionary	Model-Method	ACC	Precision	Recall	F1	BACC
1	Gabor-125-20	XGBoost-Voting	0.744	0.704	0.644	0.665	0.644
4	Gabor-125-20	LGBM-Voting	0.741	0.698	0.645	0.664	0.645
27	KSVD-250-5	LGBM-Mean	0.734	0.688	0.621	0.642	0.621
48	Gabor-125-20	XGBoost-Mean	0.729	0.676	0.613	0.632	0.613

**Table 5:** Summary of average metrics scores for each data model, class size = 15.

Position	Dictionary	Model-Method	ACC	Precision	Recall	F1	BACC
1	Gabor-250-20	LGBM-Voting	0.671	0.477	0.397	0.396	0.397
4	Gabor-250-20	XGBoost-Voting	0.668	0.451	0.39	0.387	0.390
26	KSVD-125-5	LGBM-Mean	0.658	0.45	0.379	0.382	0.379
40	KSVD-500-10	XGBoost-Mean	0.653	0.452	0.38	0.382	0.380

**Table 6:** Summary of average metrics scores for each data model, class size = 2.

Position	Dictionary	Model-Method	ACC	Precision	Recall	F1	BACC
1	Gabor-125-20	signal + coef + meta-XGBoost	0.906	0.903	0.906	0.904	0.906
5	N/A	signal-XGBoost	0.904	0.900	0.905	0.902	0.904
6	N/A	signal + meta-XGBoost	0.904	0.900	0.904	0.902	0.904
8	Gabor-125-40	signal + coef-XGBoost	0.904	0.900	0.904	0.902	0.904
12	Gabor-250-20	signal + coef + meta-LGBM	0.903	0.899	0.903	0.901	0.903
19	Gabor-250-20	signal + coef-LGBM	0.902	0.898	0.902	0.900	0.902
25	N/A	signal + meta-LGBM	0.901	0.897	0.901	0.899	0.901
32	N/A	signal-LGBM	0.900	0.896	0.900	0.898	0.900
36	Gabor-125-20	coef+meta-XGBoost	0.899	0.895	0.899	0.896	0.899
38	Gabor-125-20	coef + meta-LGBM	0.898	0.894	0.898	0.896	0.898
40	Gabor-125-20	coef-XGBoost	0.897	0.893	0.897	0.895	0.897
44	Gabor-125-20	coef-LGBM	0.896	0.892	0.895	0.893	0.895
69	N/A	meta-LGBM	0.731	0.726	0.712	0.716	0.712
70	N/A	meta-XGBoost	0.729	0.724	0.710	0.714	0.710

**Table 7:** Summary of average metrics scores for each data model, class size = 5.

Position	Dictionary	Model-Method	ACC	Precision	Recall	F1	BACC
1	Gabor-125-20	signal + coef-XGBoost	0.777	0.744	0.689	0.709	0.688
2	Gabor-250-20	signal + coef + meta-LGBM	0.777	0.745	0.690	0.711	0.690
6	N/A	signal + meta-LGBM	0.775	0.744	0.687	0.709	0.687
7	KSVD-125-20	signal + coef + meta-XGBoost	0.775	0.745	0.683	0.706	0.683
8	Gabor-250-20	signal + coef-LGBM	0.775	0.739	0.684	0.705	0.684
15	N/A	signal + meta-XGBoost	0.774	0.743	0.685	0.707	0.685
18	N/A	signal-XGBoost	0.774	0.741	0.680	0.702	0.679
23	N/A	signal-LGBM	0.773	0.741	0.684	0.706	0.684
37	Gabor-250-20	coef + meta-LGBM	0.747	0.712	0.645	0.669	0.645
38	Gabor-125-20	coef + meta-XGBoost	0.746	0.701	0.649	0.668	0.649
40	Gabor-125-20	coef-XGBoost	0.744	0.704	0.644	0.665	0.643
48	Gabor-125-20	coef-LGBM	0.741	0.698	0.645	0.664	0.645
69	N/A	meta-LGBM	0.464	0.337	0.288	0.278	0.288
70	N/A	meta-XGBoost	0.464	0.354	0.293	0.285	0.293

**Table 8:** Summary of average metrics scores for each data model, class size = 15.

Position	Dictionary	Model-Method	ACC	Precision	Recall	F1	BACC
1	Gabor-250-20	signal + coef + meta-LGBM	0.712	0.564	0.451	0.457	0.451
2	Gabor-250-20	signal + coef-LGBM	0.711	0.548	0.447	0.453	0.447
3	N/A	signal + meta-XGBoost	0.710	0.575	0.449	0.458	0.449
4	N/A	signal-XGBoost	0.709	0.594	0.448	0.456	0.448
5	Gabor-250-40	signal + coef + meta-XGBoost	0.709	0.571	0.446	0.454	0.447
6	Gabor-250-20	signal + coef-XGBoost	0.708	0.590	0.445	0.452	0.445
9	N/A	signal-LGBM	0.708	0.553	0.444	0.451	0.444
22	N/A	signal + meta-LGBM	0.706	0.530	0.443	0.449	0.443
37	Gabor-250-40	coef + meta-XGBoost	0.673	0.536	0.402	0.405	0.402
39	Gabor-250-20	coef + meta-LGBM	0.672	0.512	0.401	0.403	0.401
41	Gabor-250-20	coef-LGBM	0.671	0.477	0.397	0.396	0.397
48	Gabor-250-20	coef-XGBoost	0.668	0.451	0.390	0.387	0.390
69	N/A	meta-XGBoost	0.424	0.131	0.093	0.086	0.093
70	N/A	meta-LGBM	0.422	0.130	0.092	0.086	0.092

## Data Availability

The data presented in this study are available on request from the corresponding author.

## Appendix A

Listing A1 shows the function determining the R-wave indices for a 12-lead ECG signal.

**Listing A1.** R-wave index function for 12-lead ECG signal.

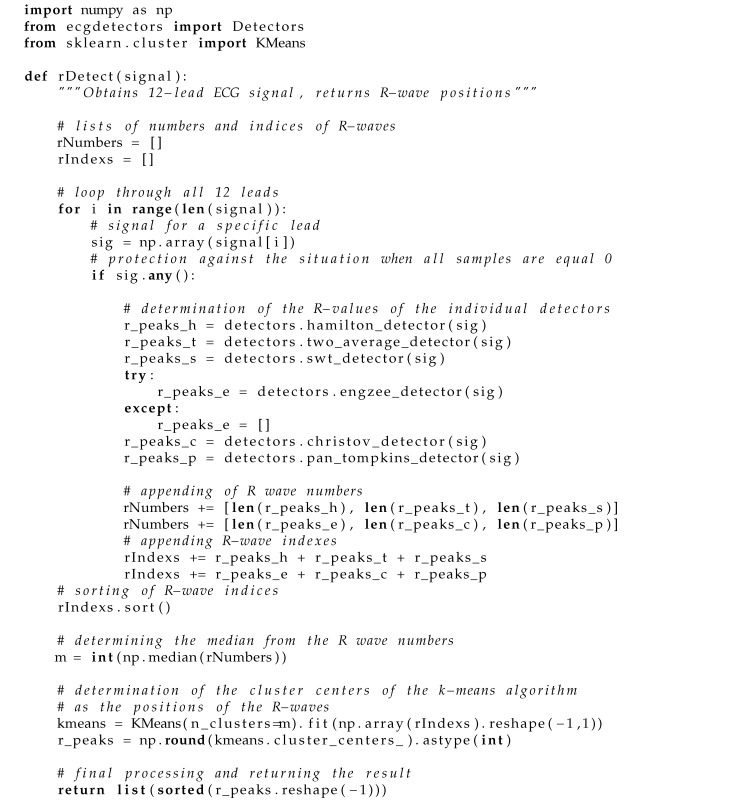

## Appendix B

Table A1 summarizes the results of the Accuracy of detection of R-wave numbers by detectors operating independently on each lead. Evaluation metrics denoted the Accuracy of this performance: MAE—mean absolute error and std—standard deviation. The abbreviations used describe the detectors studied: H—Hamilton, TA—Two Average, SWT—Stationary Wavelet Transform, Ch—Christov, PT—Pan-Tompkins, E—Engzee. The Two Average detector showed the highest Accuracy in R-wave detection, whereas the Engzee detector showed the lowest.

**Table A1:** R-peak determination Accuracy results using a one detector on a 1-lead signal.

Detector	MAE	Std
TA	0.389	0.887
SWT	0.614	1.125
PT	0.664	1.315
H	1.220	1.939
Ch	2.895	5.009
E	5.571	5.249

A novel approach using the 12-lead ECG signal was proposed to enhance the detection of R-peak. Different combinations of detectors were investigated. The best combination was evaluated using MAE and Std metrics. The results are summarized in Table A2. The Original Algorithm for the purpose of this study was a combination of Two Average detector, Christov detector, and Engzee detector.

**Table A2:** R-peak determination Accuracy results using detector combinations for 12-leads signal.

Detector	MAE	Std
TA, Ch, E	0.243	0.528
TA	0.270	0.664
H, TA, E	0.273	0.571
TA, SWT, Ch	0.297	0.570
H, TA, SWT, Ch, E	0.310	0.578
TA, Ch, PT, E	0.330	0.608
TA, PT, E	0.343	0.597
H, TA, Ch, E	0.343	0.633
H, TA, Ch, PT, E	0.350	0.637
TA, Ch, PT	0.357	0.642
TA, SWT, Ch, PT	0.360	0.633
TA, SWT, Ch, PT, E	0.367	0.622
H, TA, SWT, Ch, PT, E	0.367	0.640
H, TA, SWT	0.367	0.642
H, TA, SWT, Ch	0.377	0.644
TA, SWT, Ch, E	0.390	0.630
TA, PT	0.390	0.740
H, TA, SWT, Ch, PT	0.393	0.656
H, TA, PT, E	0.393	0.676
H, TA, SWT, PT, E	0.400	0.652
TA, SWT, PT	0.403	0.670
TA, SWT	0.410	0.668
Ch, PT, E	0.410	0.675
H, TA, PT	0.413	0.677
H, TA, SWT, E	0.417	0.655
H, TA, SWT, PT	0.420	0.682
TA, SWT, E	0.433	0.587
SWT, Ch, E	0.437	0.622
H, SWT, Ch, E	0.437	0.685
H, TA, Ch, PT	0.440	0.686
H, Ch, PT, E	0.447	0.701
TA, SWT, PT, E	0.450	0.665
H, SWT, Ch, PT, E	0.450	0.704
SWT, Ch, PT	0.453	0.715
SWT, PT, E	0.463	0.653
SWT, Ch, PT, E	0.463	0.694
H, Ch, E	0.463	0.701
H, TA	0.463	0.735
H, SWT, Ch, PT	0.483	0.726
H, PT, E	0.483	0.736
PT	0.483	0.747
H, TA, Ch	0.500	0.694
H, SWT, PT, E	0.510	0.738
SWT	0.513	0.714
SWT, PT	0.513	0.741
H, SWT, PT	0.513	0.765
H, SWT, Ch	0.517	0.729
H, SWT, E	0.527	0.765
H, Ch, PT	0.547	0.738
H, PT	0.557	0.784
H, SWT	0.563	0.839
TA, E	0.723	0.700
H, E	0.770	0.981
H	0.780	0.898
PT, E	0.810	0.827
SWT, E	0.847	0.941
TA, Ch	0.887	1.897
SWT, Ch	0.890	1.875
Ch, PT	0.990	1.939
Ch, E	1.040	1.708
H, Ch	1.323	2.351
Ch	2.483	4.474
E	4.083	3.459

## Appendix C

Table A3 shows measured computation times for classifier models.

**Table A3:** Measured computation times for classifier models for 2, 5, and 15 classes and dictionary size (ds) = 125 and 500 elements.

Model	2 cl / ds = 125	5 cl / ds = 125	15 cl / ds = 125	2 cl / ds = 500	5 cl / ds = 500	15 cl / ds = 500
KNeighbors	3 s	3 s	3 s	7 s	7 s	8 s
DecisionTree	25 s	27 s	33 s	55 s	59 s	1 min 22 s
RandomForest	27 s	32 s	37 s	34 s	37 s	43 s
SVC	8 min 15 s	12 min 43 s	12 min 29 s	33 min 43 s	55 min 22 s	1 h 1 min 9 s
XGBoost	10 s	38 s	1 min 27 s	23 s	1 min 26 s	3 min 33 s
LGBM	5 s	20 s	47 s	21 s	1 min 26 s	3 min 49 s
MLP	1 min6 s	1 min 11 s	1 min 37 s	2 min 48 s	2 min 35 s	3 min 54 s
AdaBoost	59 s	1 min 0 s	1 min 4 s	2 min 22 s	2 min 24 s	2 min 27 s
GaussianNB	1 s	2 s	3 s	6 s	8 s	12 s
